# Evaluation of moxifloxacin-induced cytotoxicity on human corneal endothelial cells

**DOI:** 10.1038/s41598-021-85834-x

**Published:** 2021-03-18

**Authors:** Joo-Hee Park, Martha Kim, Roy S. Chuck, Choul Yong Park

**Affiliations:** 1 Department of Ophthalmology, Dongguk University, Ilsan Hospital, 814, Siksadong, Ilsan-dong-gu, Goyang, Gyunggido 410-773 South Korea; 2 Department of Biochemistry, Dongguk University, College of Medicine, Gyeongju, South Korea; 3 Sensory Organ Research Institute, Dongguk University, Goyang, South Korea; 4 Department of Ophthalmology and Visual Sciences, Montefiore Medical Center, Albert Einstein College of Medicine, Bronx, NY USA

**Keywords:** Diseases, Medical research

## Abstract

Moxifloxacin hydrochloride (MXF) is widely used for the prevention of bacterial endophthalmitis after intraocular surgeries. However, the safety issue of intracameral injection of MXF for human corneal endothelial cells (HCECs) is still debatable. In this study, we investigated concentration-dependent cytotoxicity (0.05–1 mg/ml) of MXF for immortalized HCECs (B4G12 cell) and the underlying mechanism. Reactive oxygen generation (ROS) and cell viability after MXF exposure was measured. Flow cytometric analysis and TUNEL assay was used to detect apoptotic HCECs after MXF exposure. Ultrastructure of damaged HCECs by MXF was imaged by transmission electron microscope. Western blot analysis and caspase 2, 3 and 8 analysis were used to reveal the underlying mechanism of MXF induced damage in HCECs. We found that MXF induced dose-dependent cytotoxicity in HCECs. MXF exposure increased ROS generation and induced autophagy in HCECs. Increased LDH release represented the cellular membrane damage by MXF. In addition, caspases activation, Bax/Bcl-xL-dependent apoptosis pathway and apoptosis inducing factor nuclear translocation were all involved in MXF induced HCECs’ damage, especially after exposure to high dose of MXF (0.5 and 1.0 mg/ml). These findings suggest that MXF toxicity on HCECs should be thoroughly considered by ophthalmologists when intracameral injection of MXF is planned.

## Introduction

Since its introduction in the field of ophthalmology, topical moxifloxacin hydrochloride (MXF), a fluoroquinolone antibiotic, has been widely used, mainly because of its broad spectrum coverage^1^. The strong antimicrobial effect of MXF has also raised interest in its intracameral use to prevent postoperative bacterial endophthalmitis^2–4^. Other researchers are actively investigating the safety and efficacy of intracameral injection of MXF (such as Vigamox, 5 mg/ml MXF, Alcon, Fort Worth, TX)^2,3,5–7^.

Several previous studies reported the short-term in vivo and in vitro safety of intracameral injection of MXF^2,3,5,6,8^. Both lens capsular bag and anterior chamber irrigation in humans with 0.5 mg/ml of MXF, mixed with balanced salt solution, were shown to be safe for up to three months after the surgery^9^. Kernt et al*.* showed no significant toxicity of MXF exposure to human corneal endothelial cells (HCECs) up to 0.15 mg/ml for 24 h^5^. The minimum inhibitory concentration (MIC) of MXF to inhibit most of the pathogens encountered in endophthalmitis was reported to be in a range of 0.25–2.5 μg/ml^5^. However, the most common protocol for intracameral injection of Vigamox (MXF, 250 μg in 0.05 ml or 150 μg in 0.03 ml) can reach an initial anterior chamber concentration of 0.5 mg/ml, which is far above the level required for effective prophylaxis of postoperative endophthalmitis^10^.

Although the previous studies report the clinical efficacy and short-term in vivo safety of MXF intracameral injection, several researchers have questioned the safety of MXF for HCECs. HCECs are fully differentiated, and the population does not increase after birth in humans^11^. Therefore, any significant damage to HCECs can cause irreversible visual deterioration or even blindness^11^. Akal et al*.* reported the possibility of corneal oxidative damage after MXF exposure^12^. The level of oxidants and apoptosis in the entire corneal tissue increased significantly after intracameral MXF injection (0.05 mg/0.01 ml) in rat eyes^12^. In addition, Haruki et al*.* reported HCECs’ membrane damage and decreased cell viability after exposure to 0.5 mg/ml MXF^13^. Abnormal cell morphology such as giant corneal endothelial cells, was also observed in animals treated with intracameral MXF^7^.

Although there are some disagreements over the safety of intracameral MXF, there has been limited research on the mechanisms underlying possible MXF-induced corneal endothelial cell damage. In this study, we conducted an investigation into the concentration-dependent mechanisms of cell damage after MXF exposure to cultured immortalized HCECs (B4G12). We also investigated several intracellular signaling pathways related to survival and apoptosis.

## Results

### Immortalized HCECs viability and membrane damage after MXF exposure

**Figure 1:**
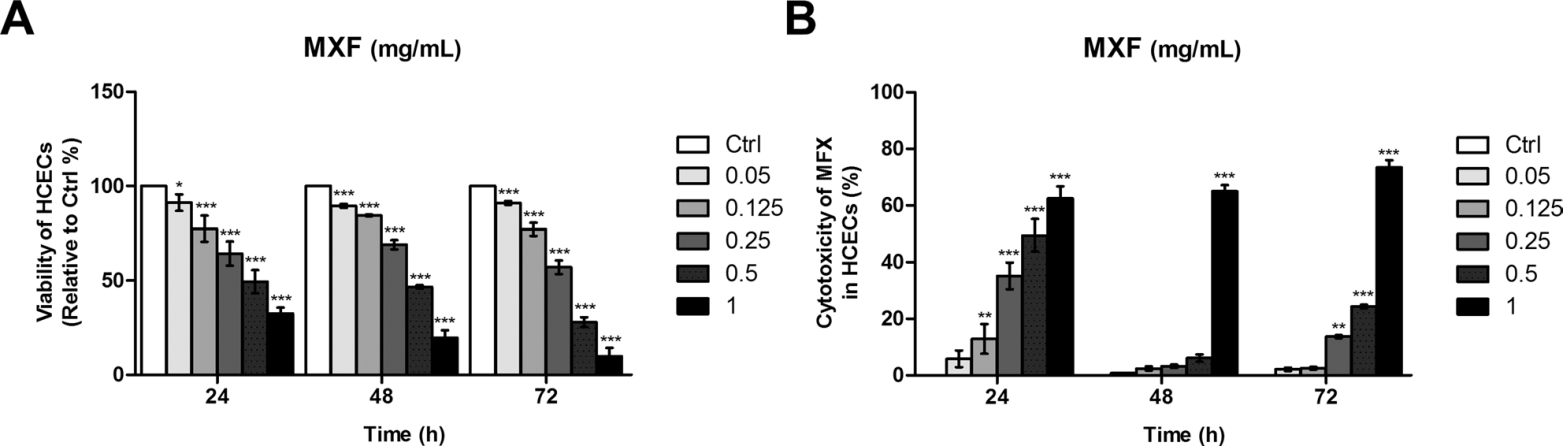
HCECs viability and toxicity assay after MXF exposure. Both cellular viability and membrane toxicity (LDH release) were measured after incubation with MXF at various concentrations (0 to 1.0 mg/ml). (**A**) MXF induced significant HCECs cytotoxicity even at 0.05 mg/ml concentration, measured after 24 h, 48 h, and 72 h of exposure. The highest MXF concentration of 1 mg/ml induced almost 85% cell death after 72 h of exposure. (**B**) The LDH release from HCECs showed a dose-dependent increase after 24 h, 48 h, and 72 h of exposure to MXF. The triplicates of each treatment group were used in each independent experiment. The values were given as the mean ± SEM from three independent experiments. (*p < 0.05; **p < 0.01; ***p < 0.001). Ctrl: no treatment control.

MXF exposure resulted in dose-dependent toxicity of immortalized HCECs (Fig. 1). After a 24-h exposure, even a low concentration of 0.05 mg/ml MXF induced a significant decrease of cell viability and a dramatic increase of LDH release. These toxicities were more prominent at longer exposure duration (from 24 to 72 h). At higher concentrations of MXF exposure (1 mg/ml), less than 20% of the cells survived after 72 h of exposure with dramatic increase of LDH release.

Vigamox is the first commercialized topical MXF ophthalmic solution. A similar toxicity pattern was observed with Vigamox treatment in immortalized HCECs (Supplementary Fig. 1). Concentrations over 0.25 mg/ml of Vigamox significantly increased cellular death from 24 to 72 h.

### Reactive oxygen species (ROS) activation by MXF

**Figure 2:**
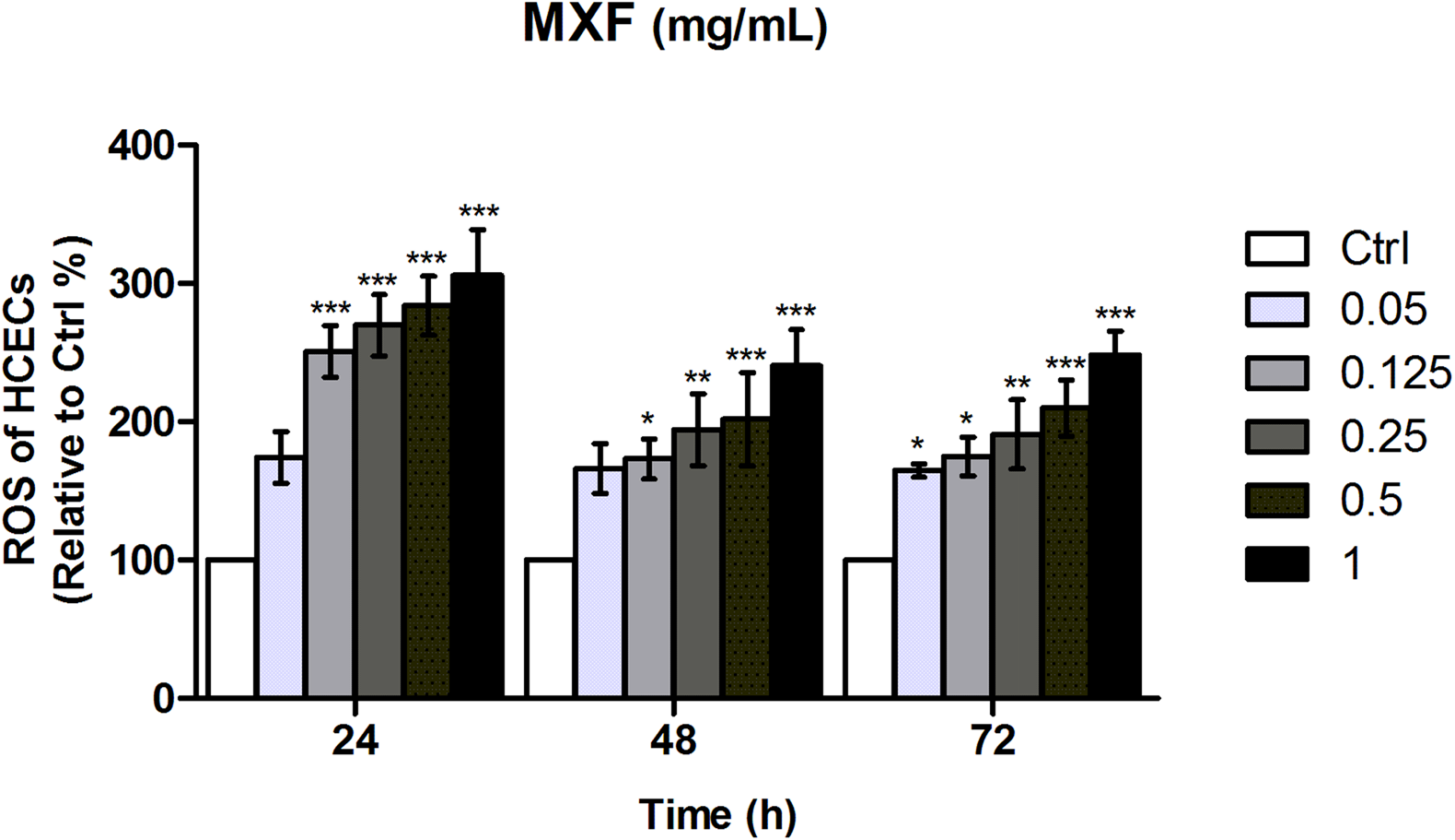
Induced reactive oxygen species (ROS) in HCECs after exposure to different concentrations of MXF for 24 h, 48 h, and 72 h. MXF increased cellular ROS generation dose dependently. Even the low concentrations of MXF (0.05 mg/ml and 0.125 mg/ml) induced significant ROS increase. The triplicates of each treatment group were used in each independent experiment. The values were given as the mean ± SEM from three independent experiments. *P* values were calculated compared with negative control. Ctrl: no treated normal control. *p < 0.05; **p < 0.01; ***p < 0.001.

MXF exposure induced significant intracellular ROS increase in immortalized HCECs both in dose- and time-dependent manners. After 24 h of incubation, a significant increase of ROS was observed, even at a low concentration of 0.125 mg/ml of MXF. The dose-dependent increase of ROS was constantly found after 48 and 72 h of exposure to MXF, and lower concentration of 0.05 mg/ml of MXF induced significant ROS increase after 72 h of exposure (Fig. 2).

### Autophagy activation by MXF

**Figure 3:**
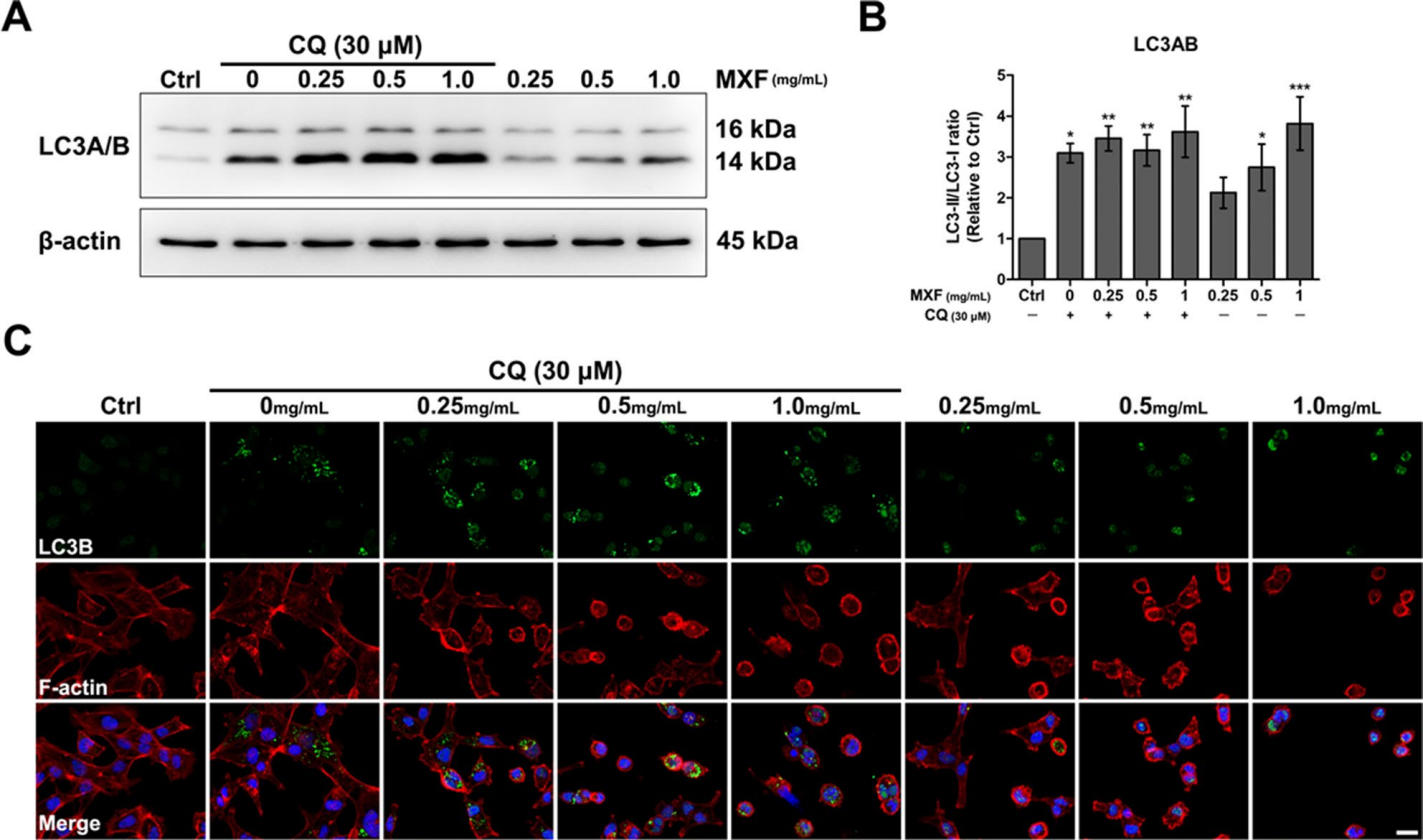
The effect of MXF on the autophagy of HCECs. MXF exposure induced a dose-dependent increase of autophagy in HCECs. LC3A/B I form (16 kDa) is converted to LC3A/B II form (14 kDa) with the activation of autophagy. As shown in (**A**) and (**B**), increased autophagy was observed after exposure to MXF. Addition of chloroquine further enhanced the LC3A/B II form formation indicating the true enhancement of autophagy by MXF. Note that only the bands at the adequate molecular weights were shown here. Full length gel and blots are included in the supplementary information. LC3B immunostaining (**C**) also verified the increased autophagy after MXF exposure. The green fluorescence (LC3B) was increased with the treatment of MXF and was further enhanced with the addition of chloroquine (30 μM). Full length electrophoresis gel image is available in supplementary files. Data shown are the representatives of three independent experiments. CQ: chloroquine, Ctrl: no treated normal control. *p < 0.05; **p < 0.01; ***p < 0.001. Scale bar: 20 μm.

Autophagy activation in immortalized HCECs was investigated by measuring LC3A/B activation. With the activation of autophagy, LC3A/B II form increases relative to LC3A/B I form. MXF exposure induced a dose-dependent increase of LC3A/B II form in HCECs (Fig. 3). Autophagy increased more than double at 0.25 mg/ml of MXF and more than threefold after 48 h of exposure to 1 mg/ml of MXF. Addition of chloroquine further enhanced LC3A/B II form indicating the increased LC3A/B II form by MXF exposure is based on the true enhanced autophagic flux.

### Ultrastructural damage of immortalized HCECs after MXF exposure

**Figure 4:**
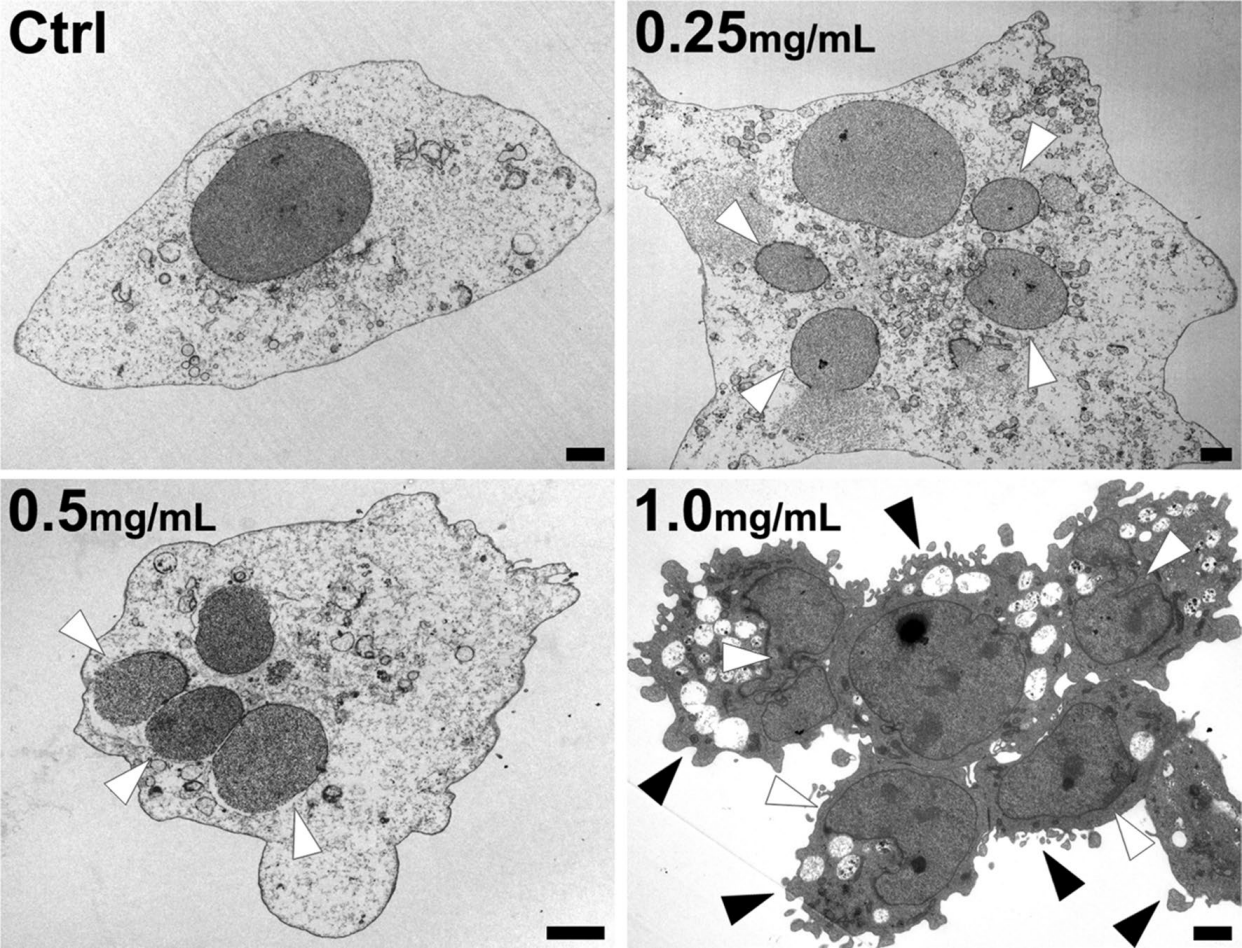
Cellular ultrastructural change of HCECs induced by MXF exposure. MXF exposure (48 h) induced significant apoptotic change of HCECs. Nuclear fragmentation (white arrowheads) was observed at 0.25 and 0.5 mg/ml of MXF exposure. At 1.0 mg/ml of MXF exposure, cytoplasmic fragmentation was also observed, indicating more severe damage to HCECs (black arrowheads). Data shown are the representatives of three independent experiments Ctrl: no treated normal control. Scale bar = 2 μm.

MXF induced both nuclear fragmentation and cytoplasmic membrane damage to immortalized HCECs in a dose-dependent manner. Nuclear fragmentation was commonly observed after exposure to 0.25 mg/ml and 0.5 mg/ml of MXF, and cytoplasmic fragmentation was detected at higher concentrations (1.0 mg/ml) of MXF exposure for 48 h (Fig. 4).

### Apoptosis assay

**Figure 5:**
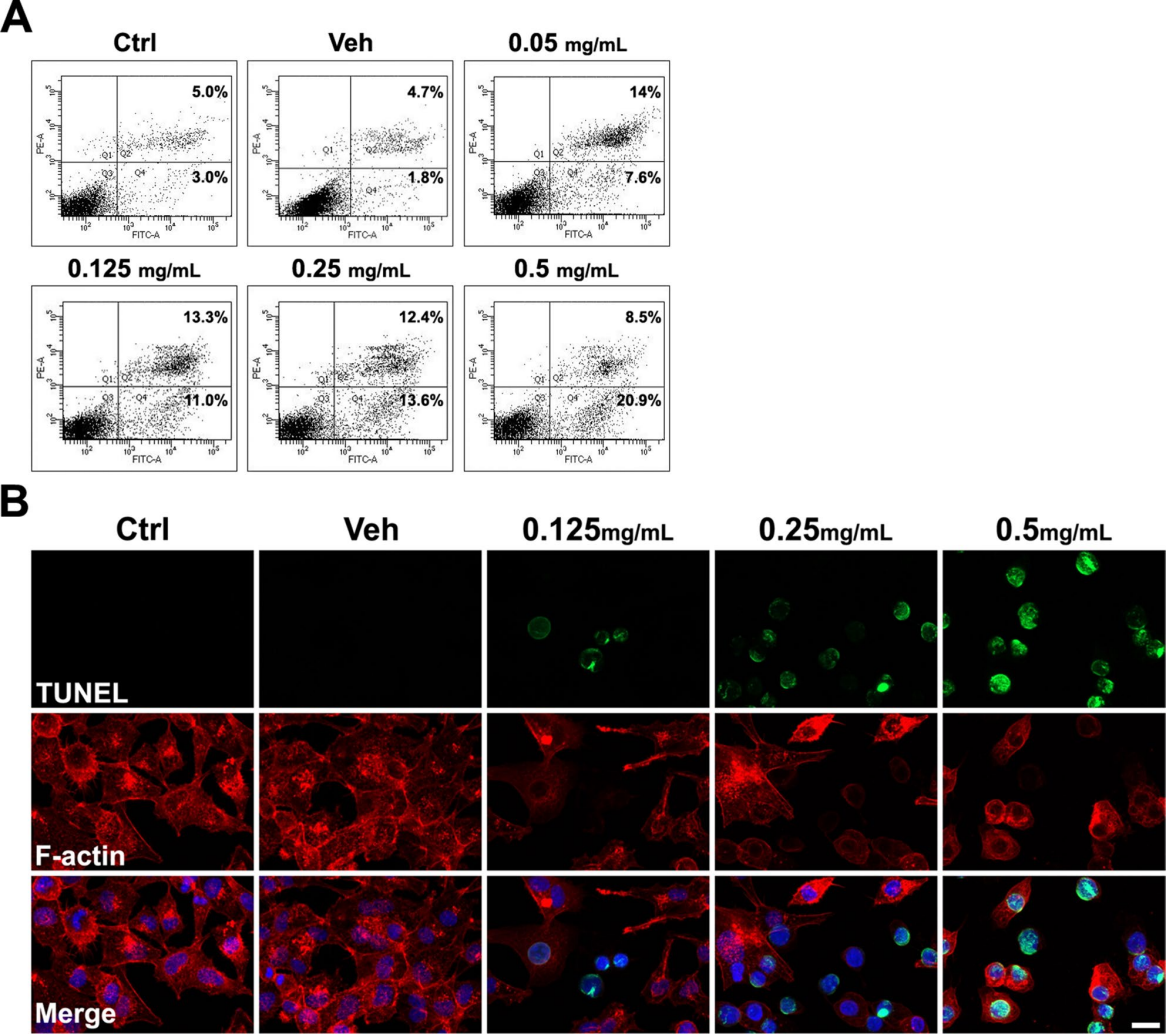
Apoptosis assay of HCECs after MXF exposure. (**A**) Flow cytometric analysis showed the increase of apoptosis (late) after exposure to MXF for 48 h. Staining with annexin V (FITC labeled) indicates early apoptosis, while staining with propidium iodide (PE labeled) indicates late apoptosis or necrosis with membrane damage. Cells in Q4 are only annexin V positive, meaning early apotosis. Cells in Q1 are only propidium iodide positive, meaning necrosis, and cells in Q2 are both annexin V and propidium iodide positive, meaning late apoptosis or early necrotic cells. A significant number of cells underwent both apoptosis and necrosis after exposure to MXF. (**B**) TUNEL-stained cells were significantly increased after high concentrations of MXF exposure compared with control. Ctrl: no treated normal control. Data shown are the representatives of three independent experiments. Veh: 0.5% DMSO.

**Figure 6:**
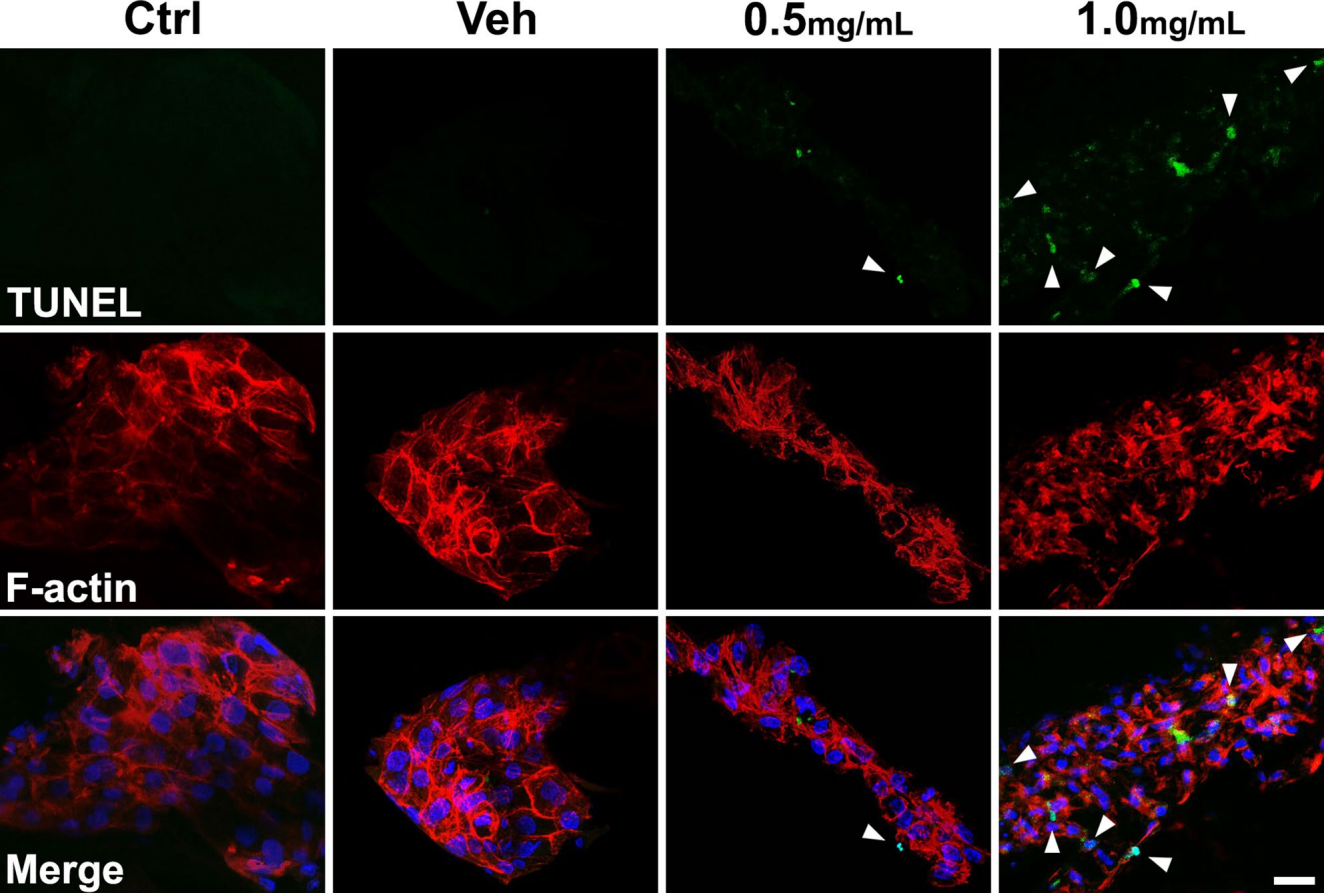
Increased apoptosis of porcine corneal endothelium after moxifloxacin exposure. Porcine corneas were stained with TUNEL after 24 h exposure to various concentrations of moxifloxacin (MXF). Compared to negative control and vehicle control, increased apoptosis of corneal endothelium was observed in corneas exposed to MXF (0.5 and 1.0 mg/mL). Green: TUNEL; Red: F-actin; Blue: DAPI. Scale bar: 20 μm.

MXF exposure also increased apoptosis of immortalized HCECs in a dose-dependent manner. Both early and late apoptosis increased, as verified by a flow cytometry (propidium iodide and annexin V) and immunocytochemical (TUNEL) staining (Fig. 5). Higher concentrations of MXF increased both late apoptotic and necrotic cell populations, compared with low concentrations of MXF. Because the totally destroyed HCECs were eliminated during the staining and flow cytometric analysis procedure, some discrepancy was inevitable between cell viability data and flow cytometric data. For example, over 50% of cell death was observed in CCK analysis (Fig. 1), while less than 30% of apoptotic and necrotic cell population in flow cytometric data after 48 h of 0.5 mg/ml of MXF exposure (Fig. 5). Induction of apoptosis of corneal endothelial cells by MXF was further verified in ex vivo porcine corneas. Incubation of corneas with MXF (0.5 and 1.0 mg mL) for 24 h resulted in apoptotic death of corneal endothelium (Fig. 6).

### Molecular evidences of MXF induced immortalized HCECs apoptosis

**Figure 7:**
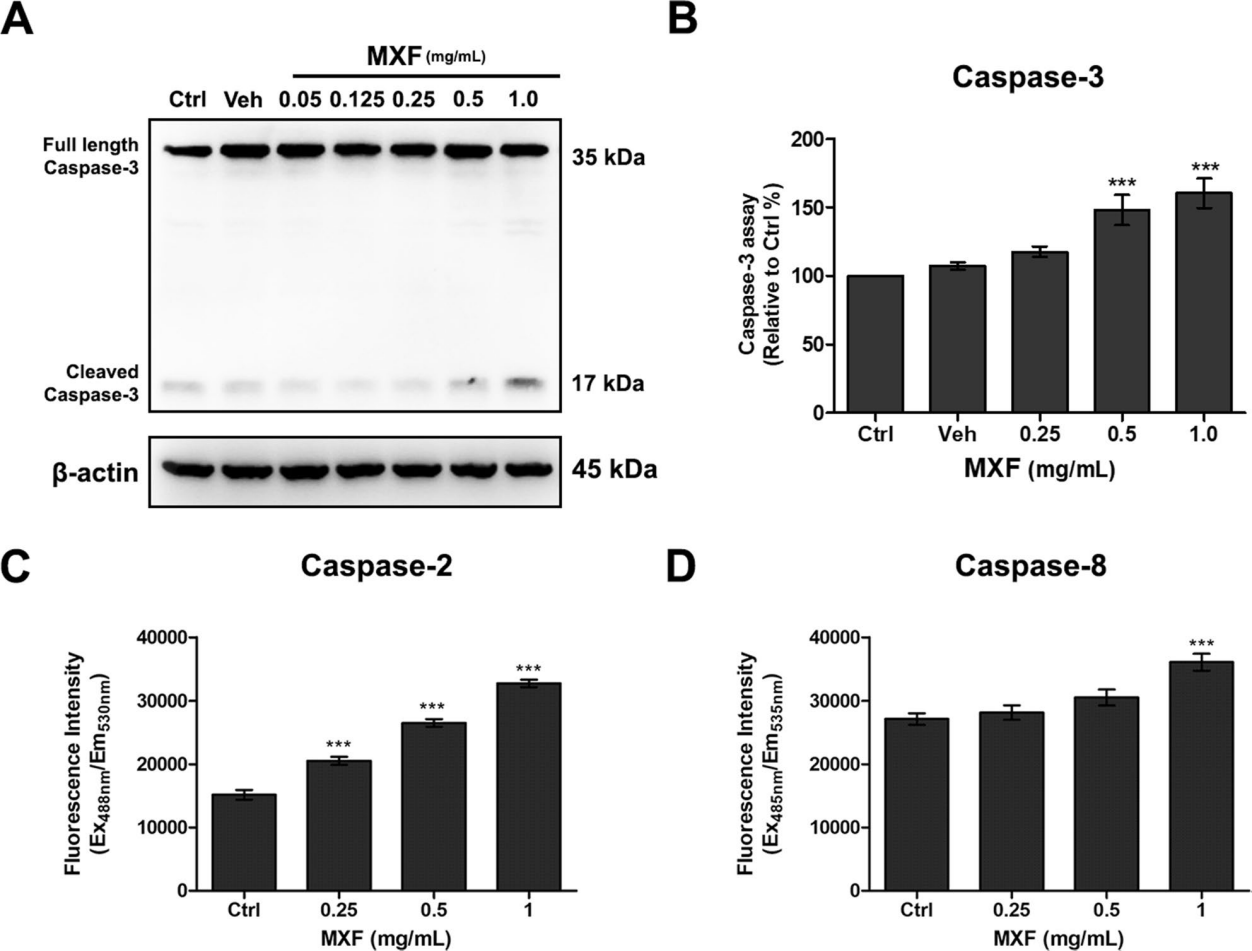
Caspase 2, 3 and 8 activation after MXF exposure in HCECs. (**A**) The activation of caspase 3 was detected after 48 h of high dose MXF exposure (0.5 and 1.0 mg/ml) Increase of cleaved form of caspase 3 on western blot analysis represents the activation of the pathway. (**B**) Caspase 3 activation after high dose MXF exposure was re-verified by caspase 3 activation analysis kit. (**C**, **D**) Caspase 2 and 8 activation after MXF exposure was verified by caspase 2 and 8 activation analysis kit. Data shown are the representatives of three independent experiments. Ctrl: no treated normal control. Veh: 0.5% DMSO. ***p < 0.001.

**Figure 8:**
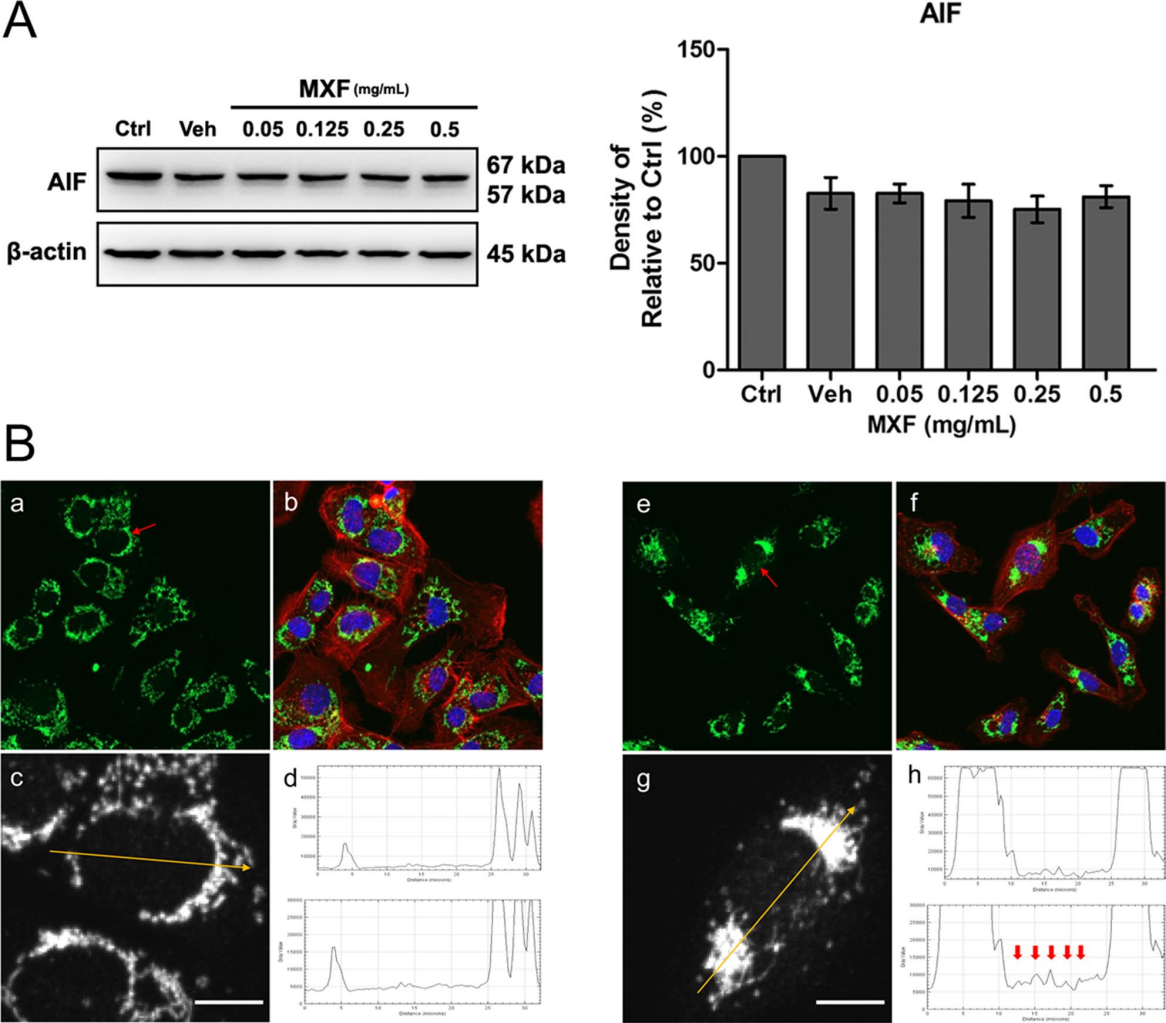
Apoptosis inducing factor (AIF) expression after MXF exposure in HCECs. (**A**) Total cellular expression level of AIF remained stable after MXF exposure. Note that only the bands at the adequate molecular weights were shown here. Full length gel and blots are included in the supplementary information. (**B**) Nuclear expression of AIF increased with MXF exposure. Panels a to d: control. Panels e to h: MXF 0.25 mg/ml exposure for 48 h; green: AIF; red: F-actin; blue: nucleus. Panels c and g are magnified gray scale images of cells (red arrow) in panels a and e. Panels d and h show the densitometry measured along the yellow arrow line in panels c and g. Red arrows in panel h indicate increased nuclear AIF signals. Full length electrophoresis gel image is available in supplementary files. Data shown are the representatives of three independent experiments. Ctrl: no treated normal control. Veh: 0.5% DMSO. Scale bar = 10 μm.

**Figure 9:**
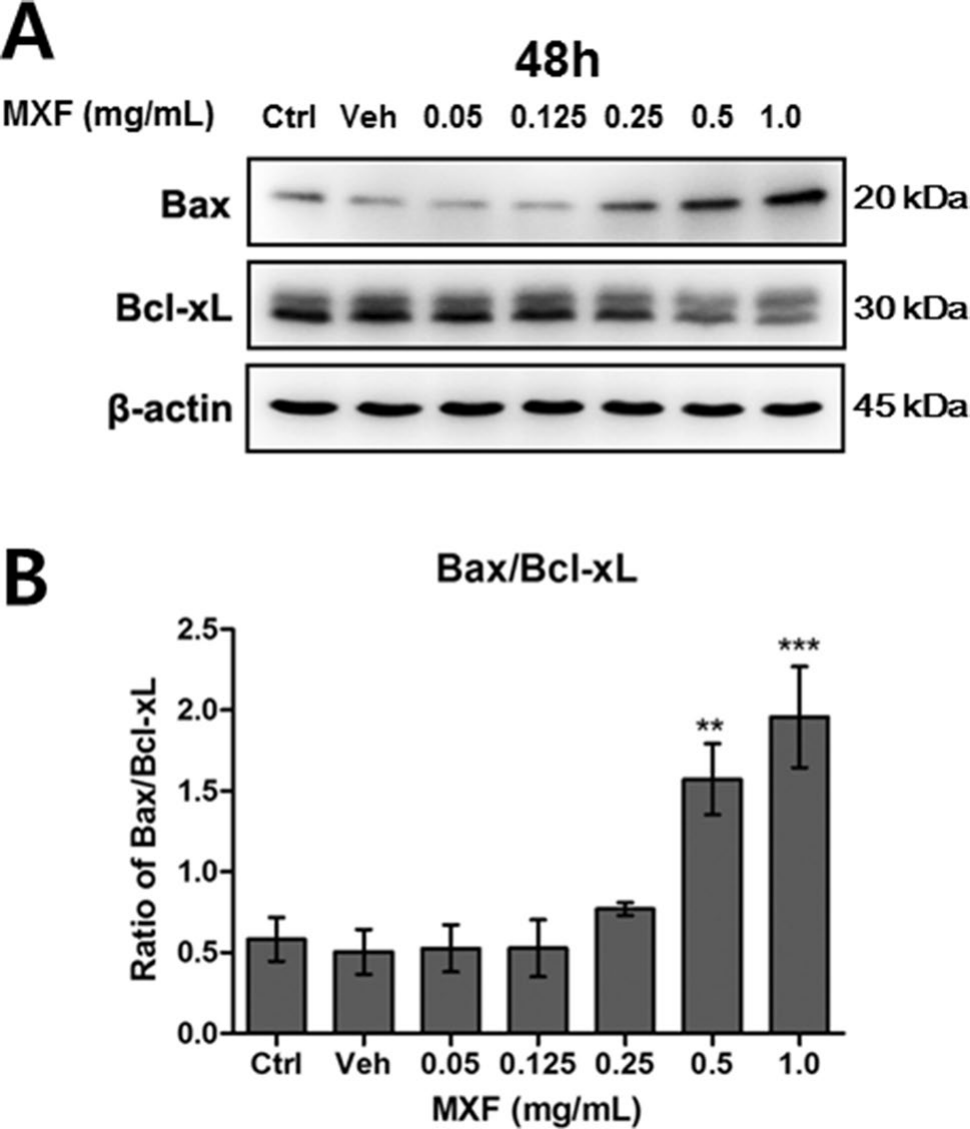
Bax/Bcl-xL ratio change by MXF exposure in HCECs. Pro-apoptotic Bax increased after MXF exposure, while anti-apoptotic Bcl-xL decreased. MXF exposure also increased Bax/Bcl-xL ratio accordingly in a dose-dependent manner. Note that only the bands at the adequate molecular weights were shown here. Full length gel and blots are included in the supplementary information. Ctrl: no treated normal control. Veh: 0.5% DMSO. *p < 0.05; **p < 0.01.

The activation of caspases 2, 3, and 8 pathways was investigated in immortalized HCECs after 48 h of exposure to MXF. Exposure to MXF resulted in significant dose dependent increases of caspase 2, 3, and 8 activation (Fig. 7). These findings indicate caspases pathway is involved in MXF induced HCECs apoptosis. AIF is normally distributed in mitochondria in the resting state and translocates to the nucleus under stress to induce apoptosis. AIF is one of the key molecules involved in a caspase-independent apoptosis pathway^14^. Although the total protein expression level of AIF was not affected, increased AIF nuclear translocation compared with control was observed after MXF exposure (Fig. 8). In addition, pro-apoptotic Bax expression increased with decreased anti-apoptotic Bcl-xL protein expression, which indicated the activation of Bax/Bcl-xL-dependent apoptosis pathway after MXF exposure (Fig. 9).

## Discussion

In this study, we found that MXF can induce immortalized HCECs death in a dose-dependent manner. The major cell damage occurred via apoptosis, combined with necrotic cell death (Supplementary Fig. 4). We verified that MXF exposure increased ROS and autophagy significantly in immortalized HCECs. The induced apoptosis by MXF involved both caspase dependent and caspase independent pathways.

We found increased LDH release from immortalized HCECs after high concentrations of MXF exposure. This finding is consistent with the previous studies^12,13^. Haruki et al*.* reported that MXF over 0.5 mg/ml caused HCEC membrane damage and death^13^. Increased oxidative stress after MXF exposure, found in our study, is also consistent with the previous studies. It is known that increased ROS can induce autophagy and eventual cell death when autophagy failed to overcome ROS^15^. Akal et al*.* reported increased oxidant content in cornea and increased caspase 3 and 8 staining in rat corneal tissue after MXF 0.05 mg/0.01 ml intraocular injection^12^. We also observed increased apoptosis of HCECs after MXF exposure. However, interestingly, our study found that MXF-induced apoptosis in HCECs was both caspase dependent and independent, which differs from the previous animal study. This difference may be due to differences in the regenerative capacity of endothelial cells. Although we used immortalized human cornea endothelial cells in the current experiment, normal HCECs do not regenerate in vivo, while corneal endothelial cells of rabbits and rats can regenerate after various forms of damage^16,17^.

The main difference between the previous studies and our current study is a more in-depth investigation of the possible mechanisms of MXF induced HCECs apoptosis. As shown previously, the increase of a cleaved form of caspase 3 (the common pathway caspase) indicates that both intrinsic and extrinsic caspase pathways are involved in MXF-induced HCECs apoptosis. The activation of caspase 8 indicates the activation of extrinsic pathway of apoptosis which is mainly mediated by the signal through the receptor on the cell membrane^15,18^. However, the activation of caspase 2 indicates the apoptosis pathway induced by the increased cellular stress such as oxidative stress, ER stress or DNA damage^15,18,19^. The finding of the activation caspase 2, 3 and 8 can be an evidence of the involvement of both intrinsic and extrinsic caspase pathways in MXF-induced HCECs’ damage. The intrinsic apoptotic pathway may also be activated by the cells if the extrinsic signal is not sufficient to execute apoptosis^18^. In addition, we found that the Bax/Bcl-xL pathways were also involved in MXF-induced HCECs apoptosis. Upregulation of the pro-apoptotic Bcl-2 family, such as Bax, and downregulation of the anti-apoptotic Bcl-2 family, such as Bcl-xL leads to caspase independent apoptosis. ^20–23^ Increased nuclear translocation of AIF can be another evidence of caspase independent apoptosis induced by high dose of MXF exposure. Our findings are similar to the previous study investigating HCEC toxicity of another ophthalmic antibiotic, norfloxacin^24^.

MXF is one of the most widely used broad spectrum topical ophthalmic antibiotics. MXF interferes with bacterial DNA gyrase and topoisomerase IV. The inhibition of either enzymes results in bacterial death. Commercial topical MXF (Vigamox) can reach anterior chamber concentrations between 1.55 and 2.28 µg/ml after 2–3 days of topical application (usually four times a day)^25^. Considering that the MIC range of MXF of most fluoroquinolone sensitive pathogens is between 0.25 and 2.5 µg/ml^26^, the topical application of MXF should be effective for the control of anterior chamber infection.

In addition, due to easy access to commercialized topical MXF formulation, some surgeons have been injecting small amounts of MXF (Vigamox) into the anterior chamber (intracameral) at the end of the cataract surgery to prevent bacterial endophthalmitis^27^, the effectiveness of which in preventing postoperative bacterial infection by MXF intracameral injection has been repeatedly reported^4,28,29^. The routine intracameral injection of MXF reduced postoperative bacterial endophthalmitis rate 3.5 to 4.0 times in a large case series (N = 617,453 and N = 116,714)^29,30^. Additionally, thorough anterior chamber irrigation, using MXF solution, has been suggested as an effective preventive action for postoperative bacterial endophthalmitis^31^.

The most common concentrations of MXF, used for intracameral injection or irrigation, are between 0.25 and 0.5 mg/ml^2,3^. Commercial MXF eyedrops, such as Vigamox, are preservative free and contain 5 mg of MXF in 1 ml of solution. Kernt et al*.* reported significant adverse effects of MXF on HCECs for concentrations higher than 0.15 mg/ml and suggested restricting intracameral Vigamox injection to concentrations less than 0.15 mg/ml^26^. Considering aqueous humor circulation system, the direct comparison between intracameral injection and 24 h culture medium exposure is impossible. However, in the current study, we found that even lower concentrations, such as 0.05 mg/ml of MXF, can induce significant ROS generation and death of HCECs as demonstrated in cell viability assay. Although the rapid clearance of moxifloxacin injected into the anterior chamber is highly expected in normal condition, the cataract surgeon should not overlook the reduced aqueous outflow, often found immediately after surgery due to retained viscoelastic device in the anterior chamber. Partial obstruction of aqueous outflow can decrease the clearance of MXF after intracameral injection and increase the exposure time of HCECs to MXF.

Our study has several limitations. Firstly, we used the HCEC cell line B4G12. Unfortunately, it is well known that the primary culture of HCECs has limited proliferation capacity. Ideally, primary-cultured HCECs from several different donors might have been better suited to our study. However, the heterogeneous population of HCECs can also cause donor-specific effects, such as age and topography-related (central vs. peripheral origin) differences^32^. For these reasons, we alternatively manipulated B4G12 instead of primary cultured HCECs in this study for the stable experiments and results because B4G12 expresses HCEC-specific 9.3. E-antigen, occludin and ZO-1 protein^33^, and shows differentiated HCECs properties in its morphology and function^34–36^. Secondly, the lack of an in vivo toxicity experiment is another drawback. Finding observed in vitro is not always repeatable in vivo because of the many confounding factors. However, HCECs toxicity study is impossible to be conducted on human eyes, because HCEC damage is irreversible and can lead to blindness. Unfortunately, convenient animal eye models, as for rabbits for example, differ from humans, in that HCECs have in vivo regeneration capability. Finally, the increased pro-apoptotic pathways were only verified after relatively high dose of MXF exposure. Therefore, the underlying mechanisms of low doses of MXF induced HCECs are still unclear. Future experiment using inhibitors or activators may enhance the evidence of the related signal pathways found in the current study.

In summary, we demonstrated a dose-dependent toxicity of MXF for immortalized HCECs. Caspases activation, Bax/Bcl-xL-dependent apoptosis pathway and AIF activation were all involved in the mechanisms of MXF toxicity for HCECs. Our results suggest that intracameral injection or irrigation of MXF should be tapered to the lowest effective concentration to avoid irreversible damage of HCECs. In addition, careful and regular postoperative monitoring of corneal endothelium is necessary after intracameral injection of MXF.

## Methods

### Human corneal endothelial cell culture

The established human corneal endothelial cells (HCECs) line, B4G12 cells (Cat no. CSC-C3457-CRA, Shirley, NY, USA), was purchased from Creative Bioarray (Shirley, NY, USA). The cells were cultured using the medium recommended by the company, which contains human endothelial serum-free medium (Creative Bioarray, Cat no. CM-345L7, Shirley, NY, USA) and 10 ng/ml of fibroblast growth factor-2 (Creative Bioarray, Cat no. CSC-CTK0134, Shirley, NY, USA). The culture medium was changed every three days, and the cells were passaged using 0.25% Trypsin–EDTA (Gibco BRL, Carlsbad, CA, USA). The cells with passage number ≤ 5 were used in this study.

### Cell viability assay

HCECs viability was measured using a commercial cell counting kit reagent (CCK-8, Dojindo Molecular Technologies, Inc. Kumamoto, Japan), according to the manufacturer’s protocol. Briefly, HCECs were cultured at 1 × 10^4^ cells/well in a 96-well plate and incubated for 24 h. MXF hydrochloride (Sigma, catalog number: SML1581) stock solution was dissolved in dimethyl sulfoxide (DMSO, Sigma, catalog number: D4540) and made serial diluted working solution. Following the adherence of cells, various concentrations (0, 0.05, 0.125, 0.25, 0.5, 1.0 mg/ml) of MXF were added to culture media for 24 h, 48 h, and 72 h respectively. After the appropriate incubation, 10 uL of CCK-8 solution was added to each cultured well, and the absorbance was measured at 450 nm after a two-hour incubation of HCECs with the reagent^37^.

### Lactate dehydrogenase (LDH) assay

Cellular membrane damage was measured using an LDH cytotoxicity detection kit (Takara Bio Inc., Shiga, Japan). Briefly, HCECs were cultured at 1 × 10^4^ cells/well in a 96-well plate and incubated for 24 h. Following the adherence of cells, various concentrations (0, 0.05, 0.125, 0.25, 0.5, 1.0 mg/ml) of MXF were applied to cells for 24 h, 48 h, and 72 h respectively. The wells with vehicle only and the wells with 1% triton X-100 addition were used as the negative and positive controls, respectively. Following the incubation of cells, all the supernatants were transferred to a new 96-well plate, the reaction mixture was added, and they were all incubated for 20 min at room temperature. Absorbance was measured at 490 nm.

### Measurement of reactive oxygen species (ROS)

Cellular ROS was measured using a ROS detection reagent (catalog number: D399: Molecular Probes, Eugene, OR, USA). HCECs were cultured at 1 × 10^4^ cells/well in a 96- well black plate and treated with different concentrations of MXF (0, 0.05, 0.125, 0.25, 0.5, 1.0 mg/ml) for 24 h, 48 h, and 72 h respectively. Following incubation, the cells were assayed for the measurement of cellular ROS, following the manufacturer’s protocol. Briefly, cell-permeant 2′,7′-dichlorodihydrofluorescein diacetate (H_2_DCFDA) was re-suspended with HCECs in the 96- well black plate, and the final working concentration was 20 μM. The plate was incubated at 5% CO_2_ 37 °C for 30 min and, finally, fluorescence was measured at 492–495 nm excitation/517–527 nm emission.

### Autophagy activation by MXF

Following the adherence of cells, various concentrations (0, 0.25, 0.5, 1.0 mg/ml) of MXF were added to culture media for 48 h. To further verify the enhancement of autophagic flux, a lysosomal protease inhibitor, chloroquine (30 μM), was used. The activation of autophagy was measured by protein expression level of LC3AB I and II forms.

### Western blot analysis

Intracellular protein expression was measured using Western blot analysis. Ice-cold radioimmunoprecipitation (RIPA) buffer [50 mM Tris–HCl (pH 8.0), 150 mM NaCl, 1% NP-40, 0.5% deoxycholate, and 0.1% sodium dodecyl sulfate (SDS)] was used for the lysis of MXF-treated HCECs for 20 min on ice. After removing the debris by centrifugation at 16,000×*g* for 10 min, total proteins were measured and quantified by Bradford assay and equal amounts (20 μg) of total cell protein were separated by SDS–polyacrylamide gel electrophoresis and transferred to a PVDF (polyvinylidene difuoride) membrane. After blocking with 3% bovine serum albumin (BSA; Sigma–Aldrich St. Louis, MO, USA) in tris-buffered saline (TBS; 10 mM Tris, pH 8.0, 150 mM NaCl) with 0.1% tween for 1 h at room temperature, the membranes were incubated overnight at 4 °C with the addition of primary antibodies: anti-apoptosis initiation factor (AIF; 1:1000; catalog number 4642S; Cell Signaling), anti-Bax (1:1000; catalog number 2772; Cell Signaling), anti-Bcl-xL (1:1000; catalog number 2764; Cell Signaling), anti-cleaved caspase 3 (1:500; catalog number 9664; Cell Signaling), anti-caspase 3 (1:1000; catalog number 9662; Cell Signaling), anti-LC3A/B (1:1000; catalog number: 12741; Cell Signaling), and mouse anti-β-actin (1:10,000; catalog number: sc-47778; Santa Cruz, Biotechnology, Dallas, Texas, USA). The membranes were then incubated with peroxidase-conjugated secondary antibodies for 1 h at room temperature. Blots were developed using an enhanced chemiluminescence kit (catalog number: RPN2232; GE healthcare, Buckinghamshire, UK) and visualized using a Fujifilm Image Reader LAS-3000 (Fujifilm, Tokyo, Japan). Each experiment was repeated at least three times, and the densitometric analysis was performed using Multi Gauge V3.0 (Fujifilm Life Science, Tokyo, Japan).

### Caspase 2, 3 and 8 assay

The activity of caspases following treatment of MXF in HCECs was measured using a commercial caspase 2 assay kit (FAM-FLICA Caspase 2 Assay Kit, ImmunoChemistry Technologies ICT-919, Bloomington, MN, USA), caspase 3 assay kit (Caspase-3 Assay Kit (colorimetric), abcam ab39401, Cambridge, UK) and caspase 8 assay kit (Caspase-8 (active) FITC Staining Kit, abcam, ab65614). According to the manufacturer’s protocol, briefly, following the adherence of HCECs, various concentrations of MXF (0, 0.25, 0.5, 1.0 mg/ml) were added to culture media for 24 h. Each MXF-treated or non-treated HCECs group was prepared at 1 × 10^6^ cellular densities for each caspase 2, 3, or 8 assays. For caspase 2 assay, the FAM-FLICA working solution was added to each trypsinized cells at a volume per volume ratio of 1:30. Following 1 h incubation at 37 °C, cells were washed and the signal was detected fluorescence intensity at Ex = 488 nm/Em = 530 nm using fluorescence microplate reader. For caspase 3 assay, cells were re-suspended in lysis buffer on ice for 10 min. After the lysis step, cytosolic extracts were collected by centrifugation and mixed with Dithiothreitol (DTT) contained reaction buffer and DEVD-chromophore p-nitroaniline (*p*-NA) substrates. DEVD is an amino acid sequence (Asp-Glu-Val-Asp) cleaved by caspase 3. The detection buffer mixed with cellular supernatants was incubated at 37 °C for 90 min and the absorbance was measured at OD 400–405 nm. For caspase 8 assay, each trypsinized cells were aliquoted, 300 µL, into microtubes. FITC-IETD-FMK was added into each tube and incubated for 1 h at 37 °C incubator (5% CO_2_). Following incubation, FITC-labeled, activated caspase 8 in HCECs was detected by fluorescence plate reader at Ex = 485 nm/Em = 535 nm.

### Annexin V FITC/PI staining for quantification of apoptosis

The late stages of cell death, resulting from either apoptosis or necrosis processes, were detected using a FITC Annexin V apoptosis detection kit II (BD Biosciences, San Jose, CA, USA), according to the manufacturer’s instructions. Briefly, MXF-treated HCECs were washed with cold DPBS and then re-suspended using 1 × binding buffer (5 × 10^5^ cells/mL); 10 μg of purified recombinant Annexin V was added in HCECs and incubated for 15 min at room temperature. Following the addition of 5 μL of FITC Annexin V and 5 μL of propidium iodide, cells were analyzed by flow cytometry (BD LSRFortessa, BD Biosciences) equipped four lasers—355 nm (UV), 405 nm (violet), 488 nm (blue), and 640 nm (red), which enables the detection of up to 18 colors simultaneously. The excitation was done at 488 nm and the emission filters used were 530 band pass (BP) for FITC labeled Annexin V, and 600 long pass (LP) for PI stained DNA. Cellular apoptosis was analysis with the BD FACSDvia software (BD Biosciences).

### Terminal deoxynucleotidyl transferase (TUNEL) assay

For the detection of fragmented DNA, due to apoptosis at the cellular level in HCECs, TUNEL assay was performed using the APO-BrdU TUNEL assay kit (catalog number: A23210: Molecular Probes), according to the manufacturer’s protocol. HCECs were seeded at a density of 6 × 10^4^ cells per milliliter and grown on Nunc Lab-Tek II chamber slides (Thermo Fisher Scientific; Waltham, MA, USA) and 0, 0.125, 0.25, and 0.5 mg/mL of MXF were treated for 24 h. The cells were fixed with 3.7% paraformaldehyde for 10 min at room temperature, and permeabilization was carried out using 0.1% triton x-100 for 5 min at room temperature. Following the washing of steps with DPBS, the cells were blocked using 1% BSA (Sigma–Aldrich) in DPBS for 30 min at RT. The chamber slides were incubated overnight at 4 °C, which were labeled using TdT enzyme and anti-BrdU mixture solution. For the final detection of the broken site of DNA, Alexa Fluor 488 dye was labeled with incorporated anti-BrdU. The staining of F-actin was carried out using fluorescent phallotoxins (1 unit; catalog number: MP00354; Molecular Probes), and the counterstaining of cell nuclei was performed using 4′,6-diamidino-2′-phenylindole (DAPI, catalog number: 10236276001; Roche Diagnostics GmbH, Mannheim, Germany). The slides were viewed using a confocal laser scanning microscope LSM800 (Carl Zeiss, Oberkochen, Germany).

### Electron microscopy analysis

The HCECs’ ultrastructural change, after MXF exposure, was investigated by transmission electron microscopy (TEM). The HCECs were treated with various concentrations of MXF (0, 0.25, 0.5, and 1.0 mg/ml) for 48 h, and then the cells were fixed in 3.7% paraformaldehyde (Sigma–Aldrich, St. Louis, MO, USA) and 2.5% glutaraldehyde (Sigma–Aldrich) in 0.1 M phosphate buffer (PB; pH 7.6) overnight. After washing in 0.1 M PB, the HCECs were fixed in 1% of osmium tetroxide in the same buffer for 1 h. Following the dehydration of the cells with a series of graded EtOH (Merck, Kenilworth, NJ, USA), HCECs were embedded in an epoxy embedding medium (Sigma–Aldrich). Then, polymerization was performed at 60 °C for three days. The ultrathin Sects. (60–70 nm) of the samples were obtained and examined under TEM (JEM-1010; JEOL, Tokyo, Japan), operating at 60 kV. The images were recorded by a charge-coupled device camera (SC1000; Gatan, Warrendale, PA, USA). The length of the electron micrograph was measured using the GMS software (Gatan).

### Ex vivo porcine corneal endothelial toxicity

Fresh pig eyes were purchased from the local slaughter house. The eyes were enucleated immediately after the death and transferred within 6 h with ice. Corneal button was harvested and cut in quadrants with a sharp surgical blade. Each segment was stained with 0.005% trypan blue mixed with minimum essential medium (MEM) for 5 min. Corneal endothelial cell viability was assessed by examining the blue-stained area under an inverted-phase contrast microscope. After the baseline viability assessment, the corneal segments were incubated with the exposure to various concentrations of MXF at 37 °C in a 5% CO_2_ and 95% air-humidified atmosphere for 24 h. The tissue culture medium was serum-free MEM containing L-glutamine (2 mM), NaHCO3 (20 g/L), penicillin (100 IE/mL), and streptomycin (0.1 mg/mL). After incubation, the corneal segments were fixed in 4% paraformaldehyde and treated in 30% sucrose. The corneal tissues were embedded in OCT compound, and then cut into 15-μm-thick horizontal sections with a cryostat. For evaluation the MXF-induced corneal apoptosis, we carried out TUNEL staining (In Situ Cell Death Detection Kit, Fluorescein; Roche Applied Science, Mannheim, Germany). According to the commercial protocol, the tissue sections were permeabilized with 0.1% Triton X-100 on ice for 2 min. and then incubated with the TdT enzyme at 37 °C for 1 h, and washed with PBS (5 min, shaking, three times). For filamentous actin staining, TRITC-conjugated phalloidin (1 µg/mL; Sigma–Aldrich) was added for 20 min and rinsed three times with PBS (5 min per rinse). The counterstaining of the cellular nucleus was performed with DAPI. The slides were examined with an LSM800 confocal laser scanning microscope, and the digital images were analyzed with ZEN lite 2012 software.

### Statistical analysis

The data were presented as mean ± standard error, and the statistical significance was determined by ANOVA and Dunnett’s multiple comparison test. *P* values of less than 0.05 were regarded as significant, according to GraphPad Prism Ver. 5.01 (GraphPad Software Inc., La Jolla, CA, USA).

## Supplementary Information

Supplementary Information

## Data Availability

The datasets generated during and/or analysed during the current study are available from the corresponding author on reasonable request.

## Abbreviations

HCEC: Human corneal endothelial cell
MXF: Moxifloxacin
ROS: Reactive oxygen species
LDH: Lactate dehydrogenase
MIC: Minimum inhibitory concentration

## References

[1] Donaldson KE, Marangon FB, Schatz L, Venkatraman AS, Alfonso EC (2006). The effect of moxifloxacin on the normal human cornea. Curr. Med. Res. Opin..

[2] Espiritu CR, Caparas VL, Bolinao JG (2007). Safety of prophylactic intracameral moxifloxacin 0.5% ophthalmic solution in cataract surgery patients. J. Cataract. Refract. Surg..

[3] Lane SS, Osher RH, Masket S, Belani S (2008). Evaluation of the safety of prophylactic intracameral moxifloxacin in cataract surgery. J. Cataract. Refract. Surg..

[4] Melega MV (2019). Safety and efficacy of intracameral moxifloxacin for prevention of post-cataract endophthalmitis: randomized controlled clinical trial. J. Cataract. Refract. Surg..

[5] Kernt M (2009). Intracameral moxifloxacin: in vitro safety on human ocular cells. Cornea.

[6] Kobayakawa S, Hiratsuka Y, Watabe Y, Murakami A, Tochikubo T (2010). Comparison of the influence of intracameral gentamicin, gatifloxacin, and moxifloxacin on the corneal endothelium in a rabbit model. Jpn. J. Ophthalmol..

[7] Carrijo-Carvalho LC, Teixeira A, de Freitas D, Carvalho FR (2016). Toxicity of intracameral injection of fourth-generation fluoroquinolones on the corneal endothelium. Cornea.

[8] Ayaki M (2010). Cytotoxicity of topical medications used for infection and inflammation control after cataract surgery in cultured corneal endothelial cells. Biocontrol Sci..

[9] Matsuura K (2014). Safety of intracameral injection of moxifloxacin using total replacement technique (bag and chamber flushing). J. Ocular Pharmacol. Therap..

[10] Asena L (2013). Ocular pharmacokinetics, safety and efficacy of intracameral moxifloxacin 0.5% solution in a rabbit model. Curr. Eye Res..

[11] Joyce NC (2003). Proliferative capacity of the corneal endothelium. Prog. Retin. Eye Res..

[12] Akal A (2015). Does moxifloxacin alter oxidant status in the cornea? An experimental study. Cutan. Ocul. Toxicol..

[13] Haruki T (2014). Comparison of toxicities of moxifloxacin, cefuroxime, and levofloxacin to corneal endothelial cells in vitro. J. Cataract Refract. Surg..

[14] Bano D, Prehn JHM (2018). Apoptosis-inducing factor (AIF) in physiology and disease: the tale of a repented natural born killer. EBioMedicine.

[15] Redza-Dutordoir M, Averill-Bates DA (1863). Activation of apoptosis signalling pathways by reactive oxygen species. Biochem. Biophys. Acta.

[16] Minkowski JS (1984). Corneal endothelial function and structure following cryo-injury in the rabbit. Invest. Ophthalmol. Vis. Sci..

[17] Tuft SJ, Williams KA, Coster DJ (1986). Endothelial repair in the rat cornea. Invest. Ophthalmol. Vis. Sci..

[18] Jin Z, El-Deiry WS (2005). Overview of cell death signaling pathways. Cancer Biol. Ther..

[19] Vigneswara V, Ahmed Z (2020). The role of caspase-2 in regulating cell fate. Cells.

[20] Kim YK (2005). Role of ERK activation in cisplatin-induced apoptosis in OK renal epithelial cells. J. Appl. Toxicol..

[21] Liu J, Mao W, Ding B, Liang CS (2008). ERKs/p53 signal transduction pathway is involved in doxorubicin-induced apoptosis in H9c2 cells and cardiomyocytes. Am. J. Physiol. Heart Circ. Physiol..

[22] Tang D (2002). ERK activation mediates cell cycle arrest and apoptosis after DNA damage independently of p53. J. Biol. Chem..

[23] Bacus SS (2001). Taxol-induced apoptosis depends on MAP kinase pathways (ERK and p38) and is independent of p53. Oncogene.

[24] Fan TJ, Wu SX, Jiang GJ (2020). Apoptotic effects of norfloxacin on corneal endothelial cells. Naunyn Schmiedebergs Arch. Pharmacol..

[25] Miller D (2008). Review of moxifloxacin hydrochloride ophthalmic solution in the treatment of bacterial eye infections. Clin. Ophthalmol..

[26] Kernt M (2010). Intracameral moxifloxacin: a safe option for endophthalmitis prophylaxis? In vitro safety profile for intraocular application. Der Ophthalmologe: Zeitschrift der Deutschen Ophthalmologischen Gesellschaft.

[27] Arshinoff SA, Modabber M (2016). Dose and administration of intracameral moxifloxacin for prophylaxis of postoperative endophthalmitis. J. Cataract. Refract. Surg..

[28] Lucena NP (2018). Intracameral moxifloxacin after cataract surgery: a prospective study. Arq. Bras. Oftalmol..

[29] Haripriya A, Chang DF, Ravindran RD (2017). Endophthalmitis reduction with intracameral moxifloxacin prophylaxis: analysis of 600 000 surgeries. Ophthalmology.

[30] Haripriya A, Chang DF, Namburar S, Smita A, Ravindran RD (2016). Efficacy of intracameral moxifloxacin endophthalmitis prophylaxis at aravind eye hospital. Ophthalmology.

[31] Matsuura K, Suto C, Akura J, Inoue Y (2013). Bag and chamber flushing: a new method of using intracameral moxifloxacin to irrigate the anterior chamber and the area behind the intraocular lens. Graefe's Arch. Clin. Exp. Ophthalmol..

[32] Joyce NC (2012). Proliferative capacity of corneal endothelial cells. Exp. Eye Res..

[33] Valtink M, Gruschwitz R, Funk RH, Engelmann K (2008). Two clonal cell lines of immortalized human corneal endothelial cells show either differentiated or precursor cell characteristics. Cells Tissues Organs.

[34] Sornelli F, Lambiase A, Mantelli F, Aloe L (2010). NGF and NGF-receptor expression of cultured immortalized human corneal endothelial cells. Mol. Vis..

[35] Hsueh YJ (2015). Lysophosphatidic acid induces YAP-promoted proliferation of human corneal endothelial cells via PI3K and ROCK pathways. Mol. Ther. Methods Clin. Dev..

[36] Levis HJ (2012). Plastic compressed collagen as a novel carrier for expanded human corneal endothelial cells for transplantation. PLoS ONE.

[37] Kim JY (2017). Safety of nonporous silica nanoparticles in human corneal endothelial cells. Sci. Rep..

