# Supplementary material for: Evaluation of moxifloxacin-induced cytotoxicity on human corneal endothelial cells
Source: Sci Rep. 2021 Mar 18;11:6250. doi: 10.1038/s41598-021-85834-x (PMC7973544; doi:10.1038/s41598-021-85834-x)

Evaluation of moxifloxacin-induced cytotoxicity on human corneal endothelial cells

Joo-Hee **Park** PhD^ab^, Martha **Kim** MD^ac^, Roy S. **Chuck** MD, PhD^d^, Choul Yong **Park** MD, PhD^ac^

1. Department of Ophthalmology, Dongguk University, Ilsan Hospital, Goyang, South Korea
2. Department of Biochemistry, Dongguk University, College of Medicine, Gyeongju, South Korea
3. Sensory Organ Research Institute, Dongguk University, Goyang, South Korea
4. Department of Ophthalmology and Visual Sciences, Montefiore Medical Center, Albert Einstein College of Medicine, Bronx, NY, USA

Joo-Hee Park ([asalella00@gmail.com](mailto:asalella00@gmail.com)), Martha Kim ([marthakim22@gmail.com](mailto:marthakim22@gmail.com)), Roy S. Chuck ([rchuck@montefiore.org](mailto:rchuck@montefiore.org)), Choul Yong Park (oph0112@gmail.com),

*Correspondence to:

Choul Yong Park MD, PhD

Department of Ophthalmology

Dongguk University, Ilsan Hospital

814, Siksadong, Ilsan-dong-gu, Goyang

Gyunggido, South Korea, 410-773

Tel: 82-31-961-7395

Fax: 82-31-961-7977

Email: [oph0112@gmail.com](mailto:oph0112@gmail.com)

**Supporting information**

**Supplementary Figure 1**. **HCECs viability after exposure to Vigamox^TM^.**

Cellular viability was measured after incubation with various concentrations of Vigamox^TM^ (0 to 2.0 mg/ml). Dose-dependent cytotoxicity was prominent in HCECs after Vigamox^TM^ exposure for 24 h, 48 h, and 72 h. The triplicates of each treatment group were used in each independent experiment. The values were the mean ± SEM from three independent experiments. * *p* < 0.05, ** *p* < 0.01, *** *p* < 0.001.


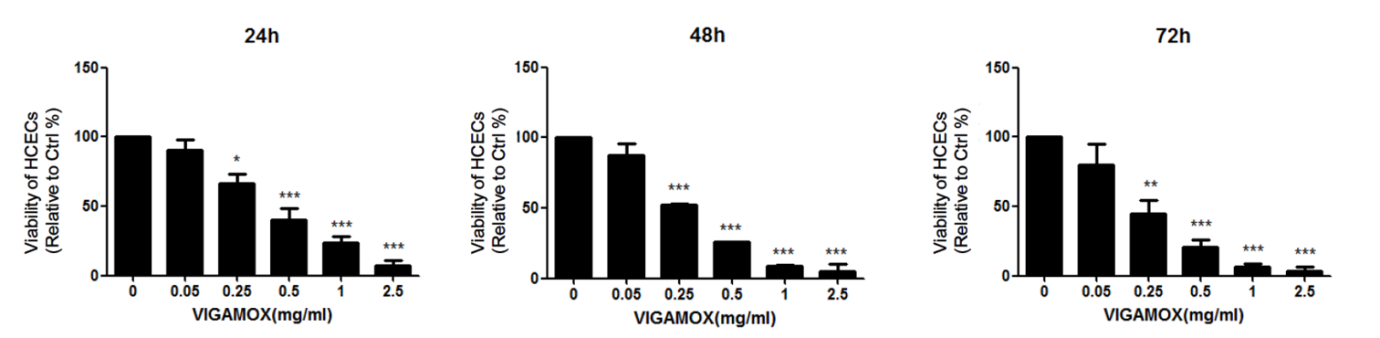


**Supplementary Figure 2**. **HCECs viability and LDH assay after exposure to DMSO.**

Cellular viability and LDH were measured after incubation with DMSO of various concentrations (0 to 2.0 mg/ml). DMSO is a solvent used to dissolve moxifloxacin hydrochloride. No significant HCECs cytotoxicity was observed after DMSO exposure up to 1 mg/mL for 72 h. LDH showed some increase after DMSO exposure but failed to reach statistical significance.


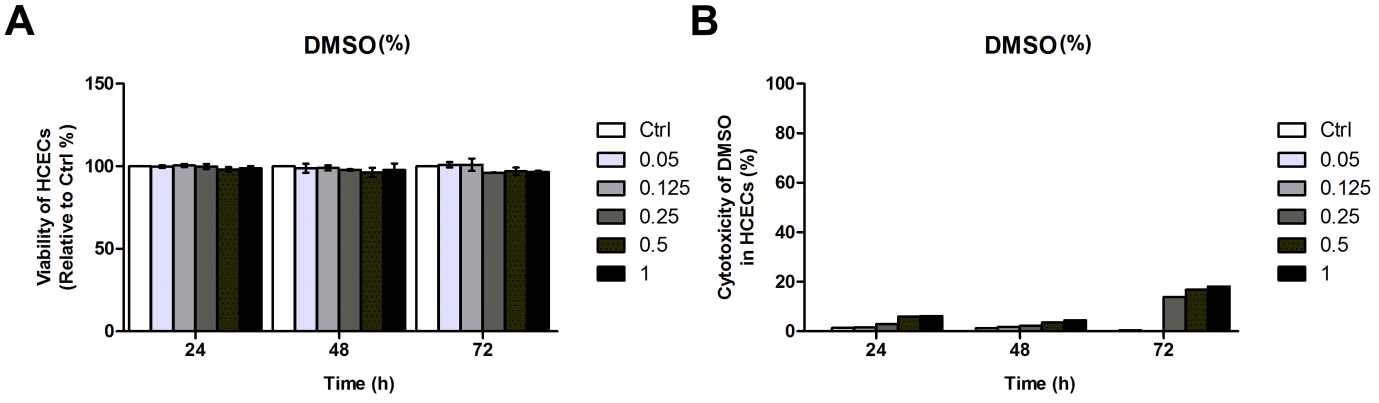


**Supplementary Figure 3. Autophagosome and autolysosome found in B4G12 cells after moxifloxacin exposure.**

Autophagosome and autolysosome found in B4G12 cells after 48h-exposure to moxifloxacin (0.5mg/mL) imaged by an electron microscope. The black arrowhead indicates doubled, even tripled membraned vesicles known as autophagosome and the white arrowhead represents autolysosome which is autophagosome fused with lysosome. Higher magnified figures were in boxed.


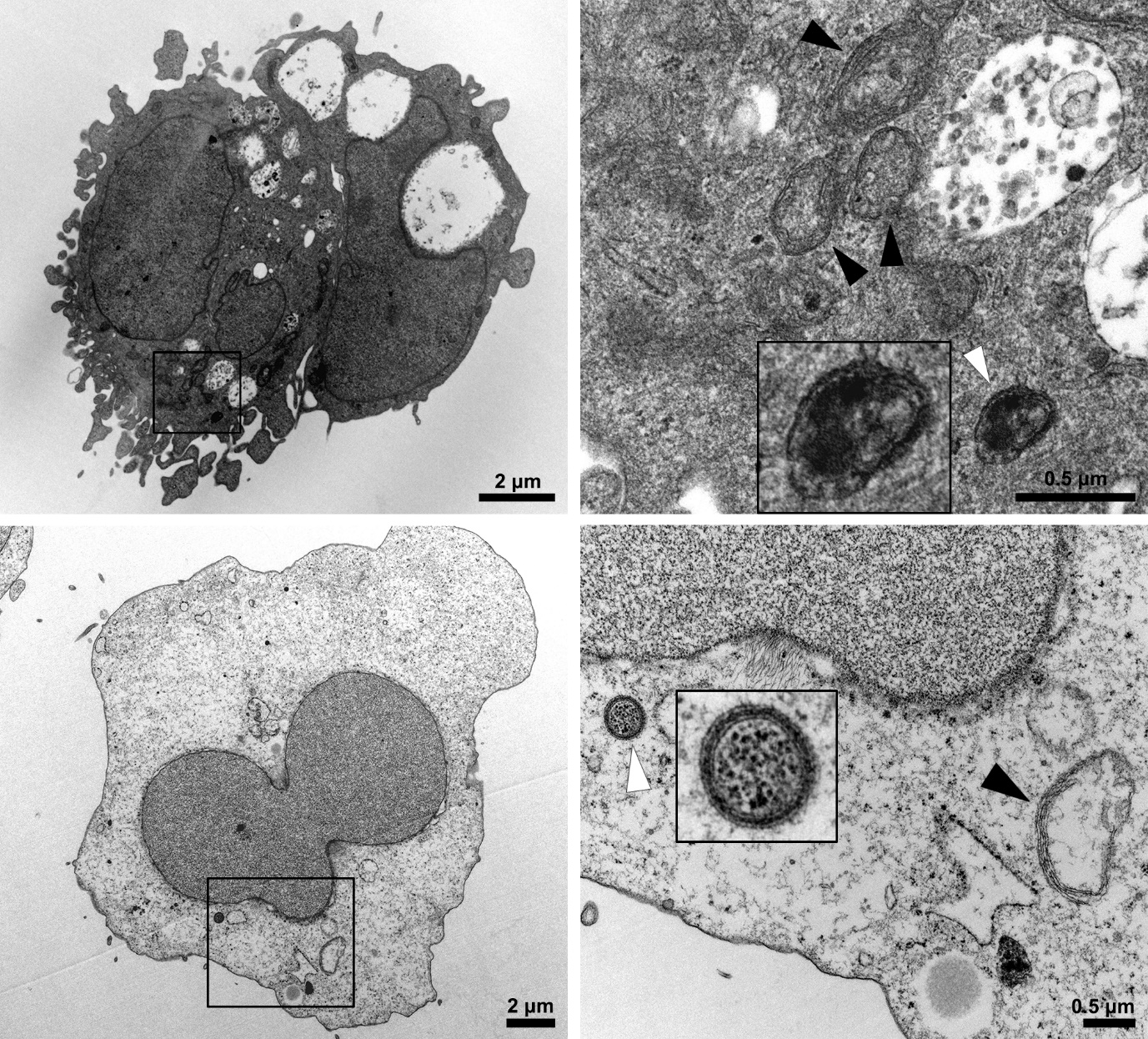


**Supplementary Figure 4. MXF induced necrosis in B4G12 cells.**

We could observe damages of plasma membrane in B4G12, 48h after treatment of MXF (1mg/mL) using TEM. Cellular components were flowed out from the burst cell which is necrosis phenomenon of the cell (Fink and cookson *et al*., Infection and Immunity, 2005 (73) 4; 1907–1916; Apoptosis, Pyroptosis, and Necrosis: Mechanistic Description of Dead and Dying Eukaryotic Cells).


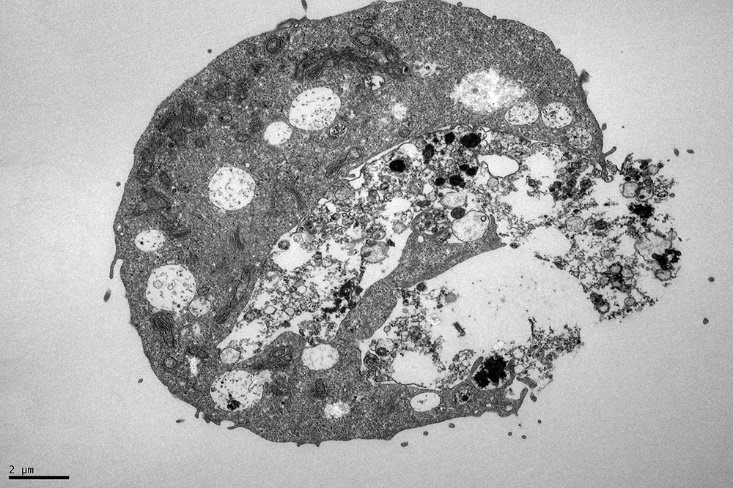


**Full length, uncropped images and cropped area of immunoblot**

Figure 3.

LC3A/B


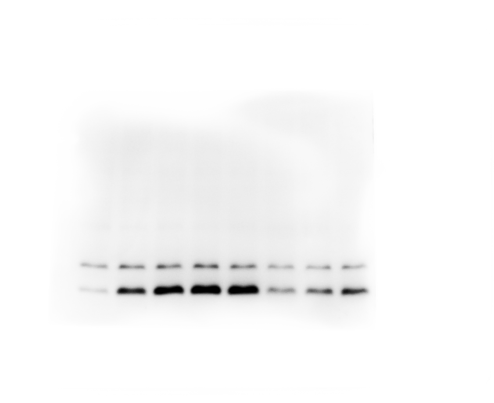

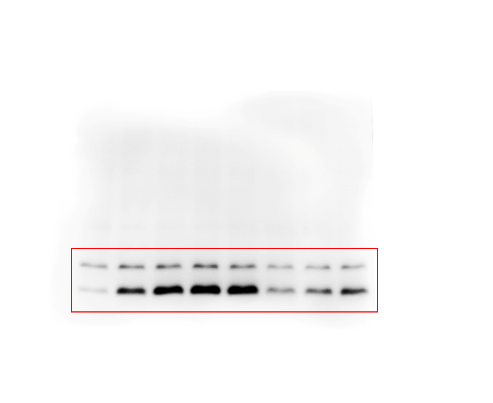


Beta actin





Figure 7.

Caspase 3


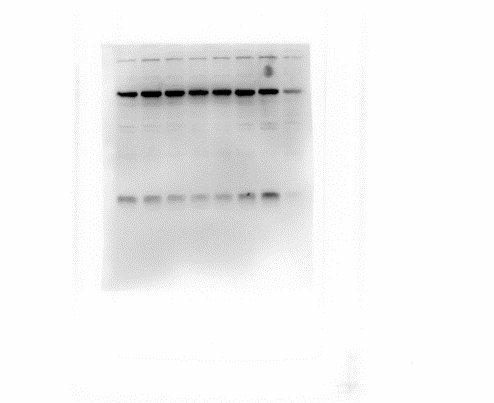

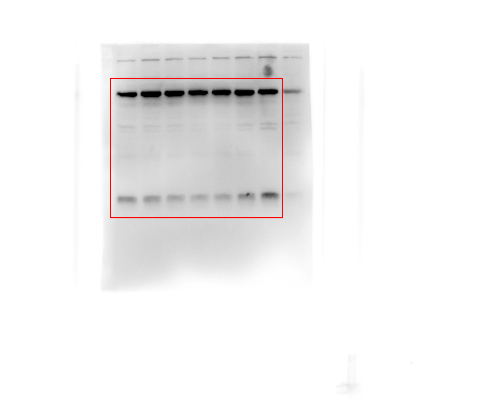


Beta actin


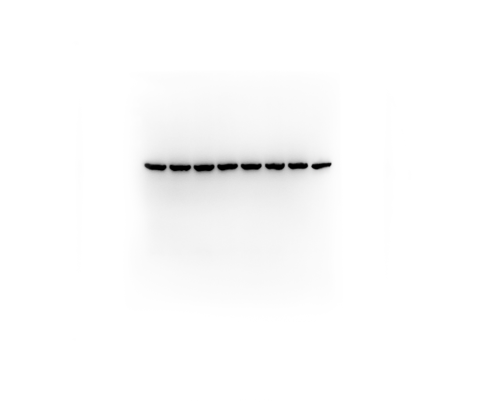

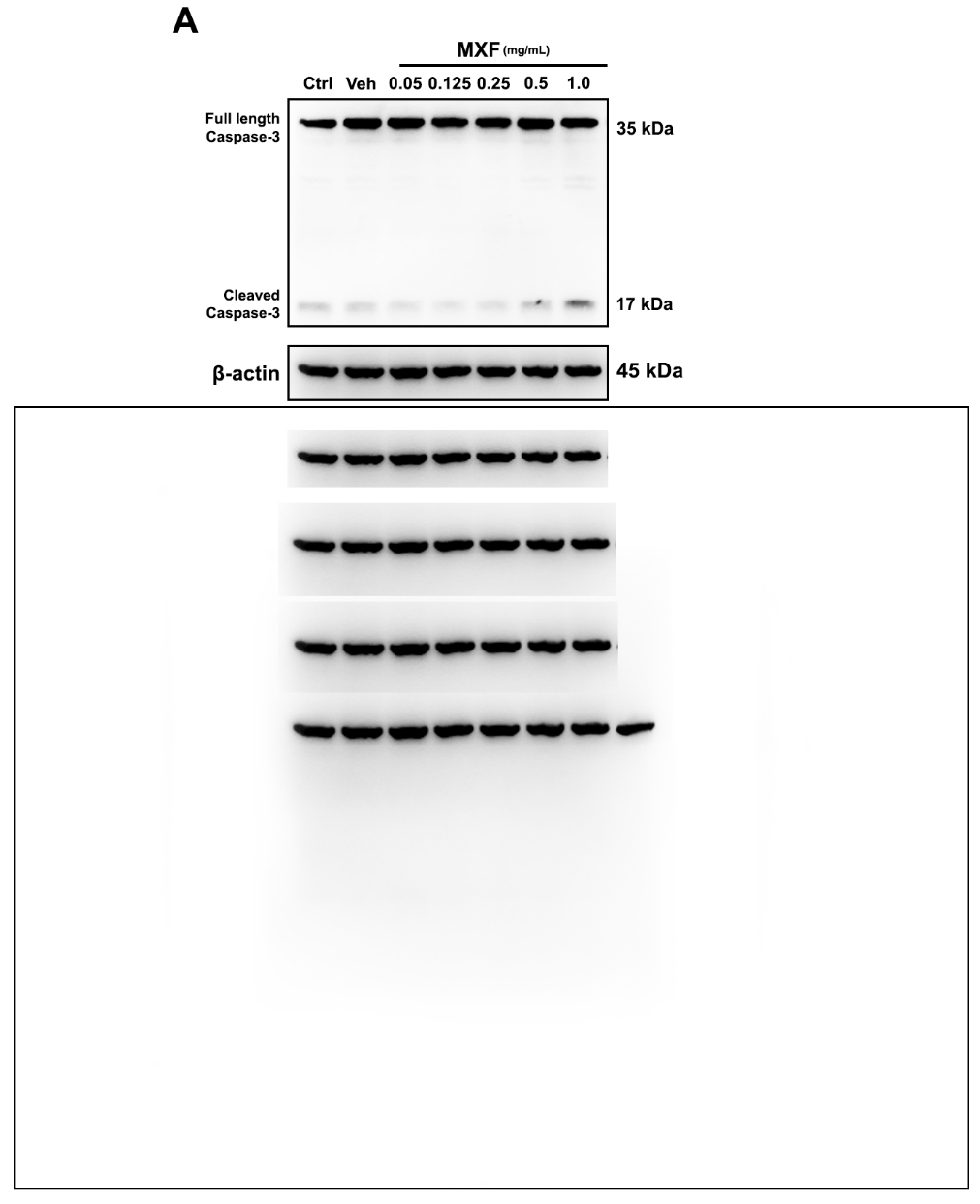


Figure 8.

AIF


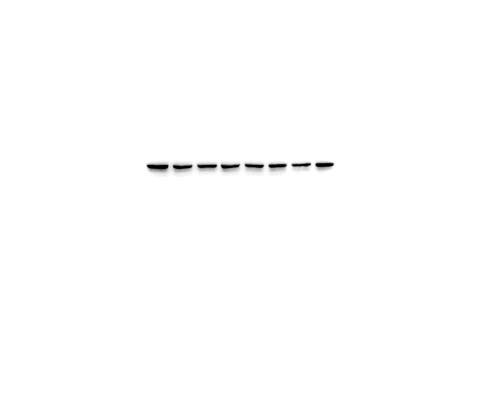

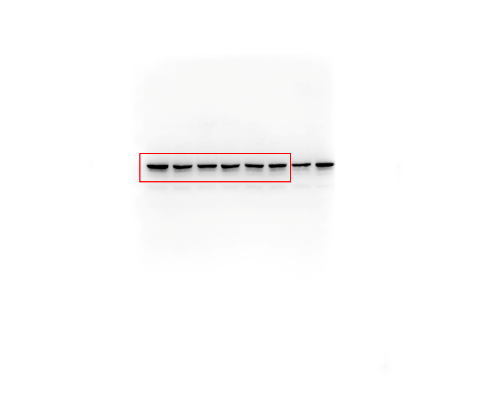


Beta actin


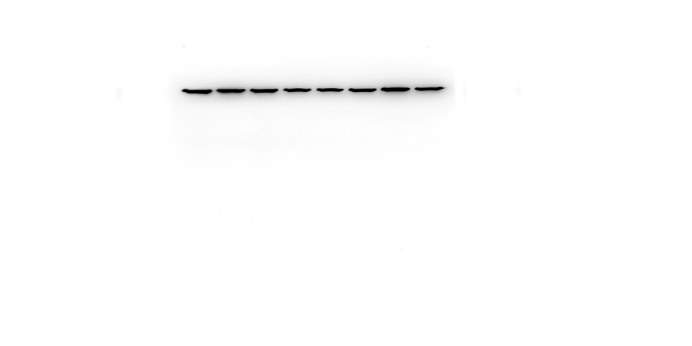


Figure 9.

BAX


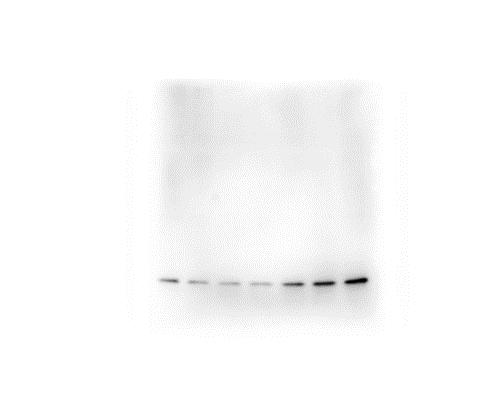

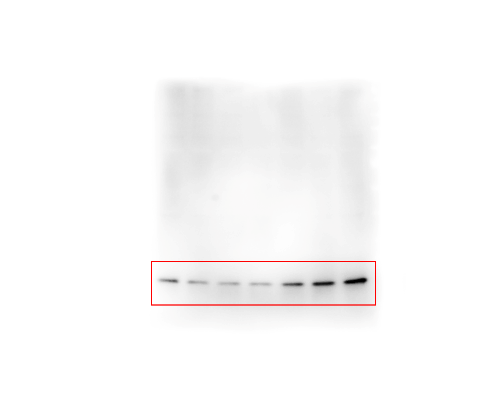


[^1^](#_ENREF_1)Bcl-XL


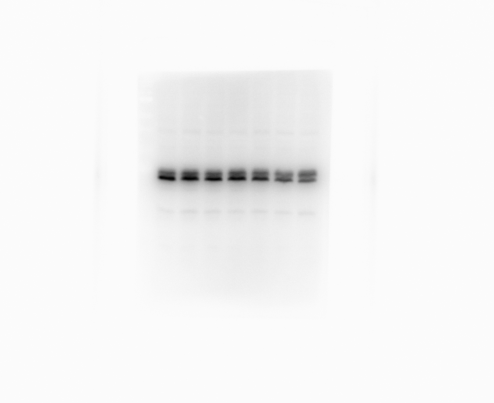

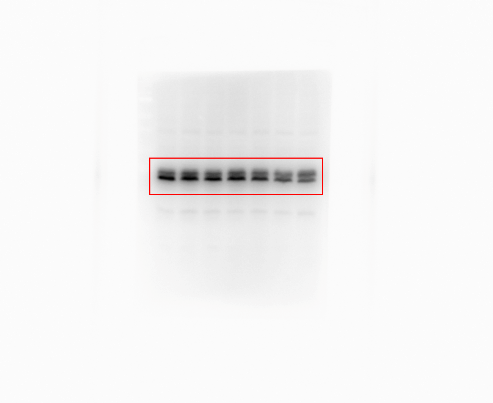


Beta actin


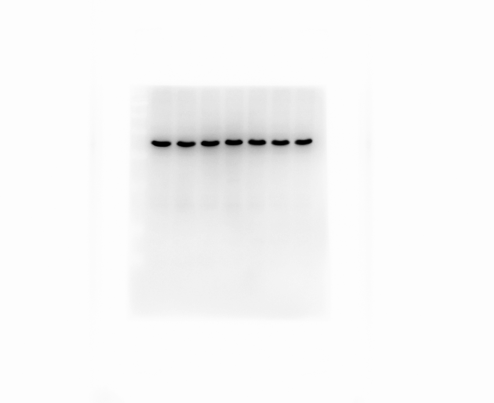

Supplement: Supplementary file 1 — Supplementary Information [file 41598_2021_85834_MOESM1_ESM.docx]
